# Supplementary material for: A robust method for measuring aminoacylation through tRNA-Seq
Source: bioRxiv. 2024 Mar 6:2023.07.31.551363. Originally published 2023 Aug 1. Preprint. [Version 2] doi: 10.1101/2023.07.31.551363 (PMC10418082; doi:10.1101/2023.07.31.551363)
Supplement: 6 [file NIHPP2023.07.31.551363V2-supplement-1.pdf]

bioRxiv preprint doi: https://doi.org/10.1101/2023.07.31.551363; this version posted March 6, 2024. The copyright holder for this preprint (which was not certified by peer review) is the author/funder, who has granted bioRxiv a license to display the preprint in perpetuity. It is made available under aCC-BY 4.0 International license.

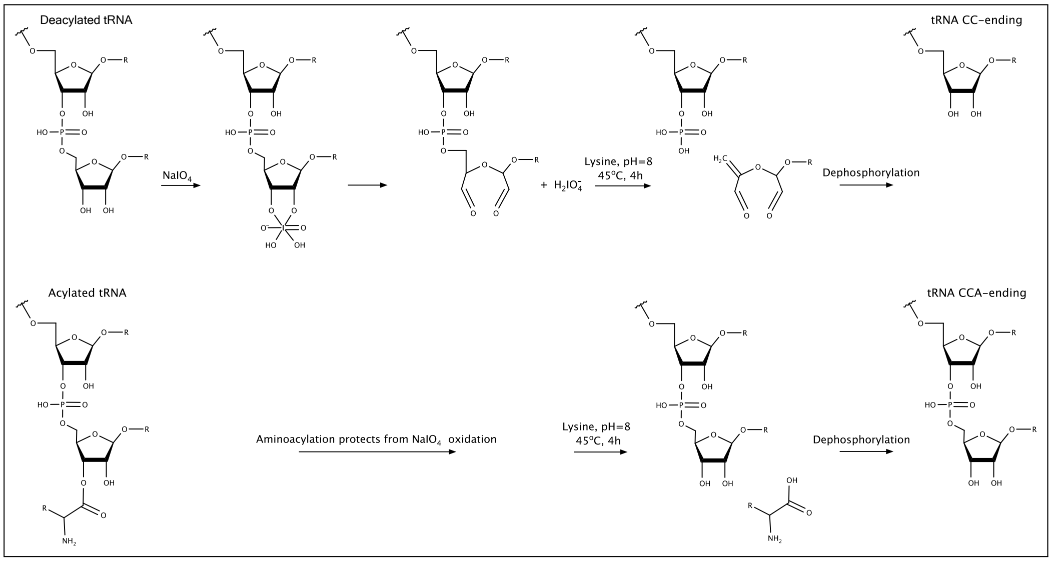

Figure 1—figure supplement 1. Schematic of the Whitfeld reaction with acylated and deacylated tRNA leading to generation of CCA and CC-ending tRNAs. For deacylated tRNA, 3′ adenosine is oxidized by periodate and then cleaved off by lysine induced β-elimination (***Rammler, 1971***; ***Uziel, 1973***). Acylated tRNA is protected from periodate oxidation but will be deacylated in the subsequent incubation with lysine.

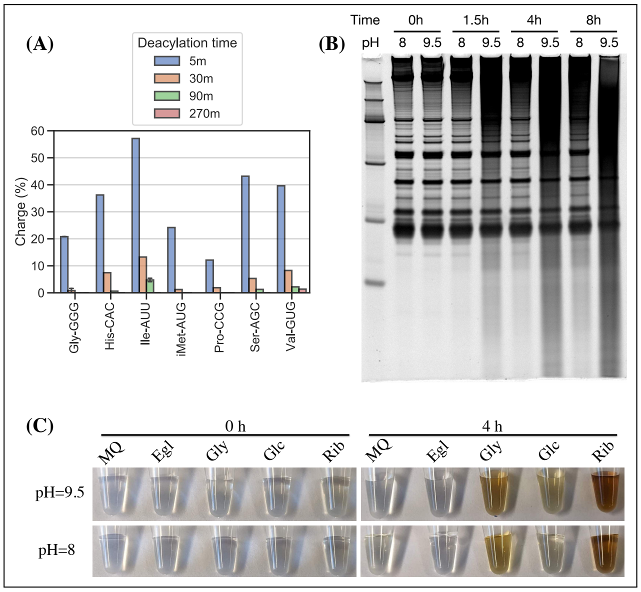

Figure 2—figure supplement 1. Optimizing lysine induced cleavage for the charge tRNA-Seq method. **(A)** Aminoacylation remaining after 5, 30, 90 and 270 min of deacylation in 1 M lysine pH=8 at 45°C. After deacylation, RNA was purified and submitted to the Whitfeld reaction using lysine cleavage at pH=9.5 for 90 min at 45°C to ensure complete deacylation. The RNA was then processed using the described charge tRNA-Seq method. **(B)** RNA stability over time for lysine cleavage at pH=8 and borax cleavage at pH=9.5. **(C)** Lysine reacts with dialdehydes forming from quencher oxidation. One-pot Whitfeld reactions were performed at pH=8 and pH=9.5 and quenched with either water (MQ), ethylene glycol (Egl), glycerol (Gly), glucose (Glc) or ribose (Rib). Pictures taken before (0 h) and after (4 h) the lysine cleavage step indicate side product formation consistent with lysine reacting with dialdehydes formed during the periodate quenching (***Saraiva et al., 2006***). This side product causes problems in the later purification step.

bioRxiv preprint doi: https://doi.org/10.1101/2023.07.31.551363; this version posted March 6, 2024. The copyright holder for this preprint (which was not certified by peer review) is the author/funder, who has granted bioRxiv a license to display the preprint in perpetuity. It is made available under aCC-BY 4.0 International license.

833

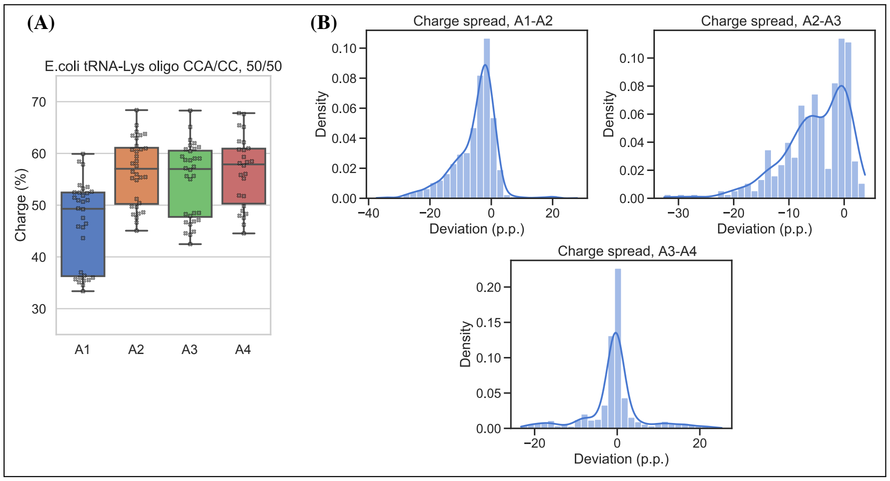

Figure 2—figure supplement 2. Measurement bias in charge tRNA-Seq using blunt-end ligation. **(A)** Measured charge of a E.coli tRNA-Lys oligo control spiked into samples processed with four different pre-adenylated adapters. The control was made using a mix of 50% E.coli tRNA-Lys-CCA and 50% E.coli tRNA-Lys-CC and thus simulating 50% charge. Each dot represents a single charge tRNA-Seq sample. **(B)** Distribution of charge differences at the transcript level among samples with two barcode replicates, comparing adapters A1 vs. A2, A2 vs. A3 and A3 vs. A4. Deviation is reported as percentage point differences and the kernel density estimate (KDE) is overlaid.

834

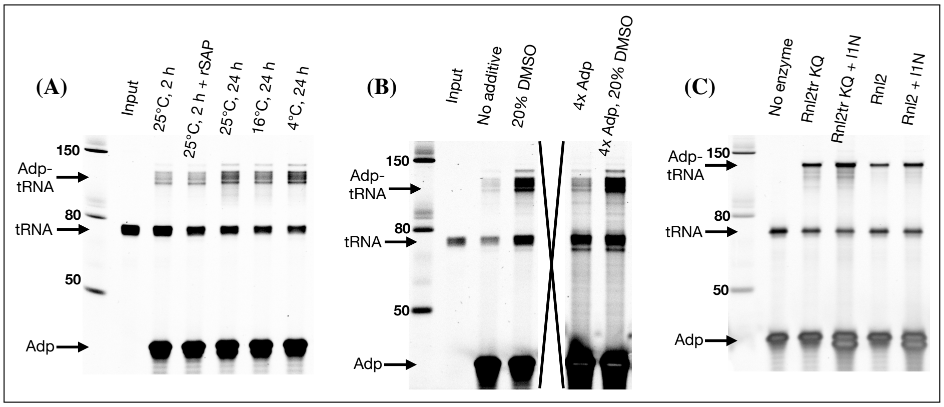

Figure 2—figure supplement 3. Despite optimization attempts, high ligation efficiency could not be achieved for blunt-end ligation. **(A)** Effect of incubation temperature, time and addition of a phosphatase (rSAP). Using deacylated and gel purified human tRNA as substrate and pre-adenylated l3N as adapter, otherwise following the blunt-end ligation method described in the methods section. **(B)** Effect of additives and higher adapter concentration. Using deacylated and gel purified human tRNA as substrate, pre-adenylated l2N as adapter and 4°C, 24 h incubation. An irrelevant well has been crossed out to avoid image splicing. **(C)** Effect of ligase type. Using the E.coli tRNA-Lys-CCA oligo as substrate, pre-adenylated l1N as adapter and 4°C, 24 h incubation with 20% DMSO. Wells with "+l1N" were added additional none pre-adenylated adapter. Rnl2tr KQ (T4 RNA Ligase 2, truncated KQ) is the standard ligase used for pre-adenylated adapters whereas Rnl2 (T4 RNA Ligase 2) does not require pre-adenylation of adapters.

bioRxiv preprint doi: https://doi.org/10.1101/2023.07.31.551363; this version posted March 6, 2024. The copyright holder for this preprint (which was not certified by peer review) is the author/funder, who has granted bioRxiv a license to display the preprint in perpetuity. It is made available under aCC-BY 4.0 International license.

835

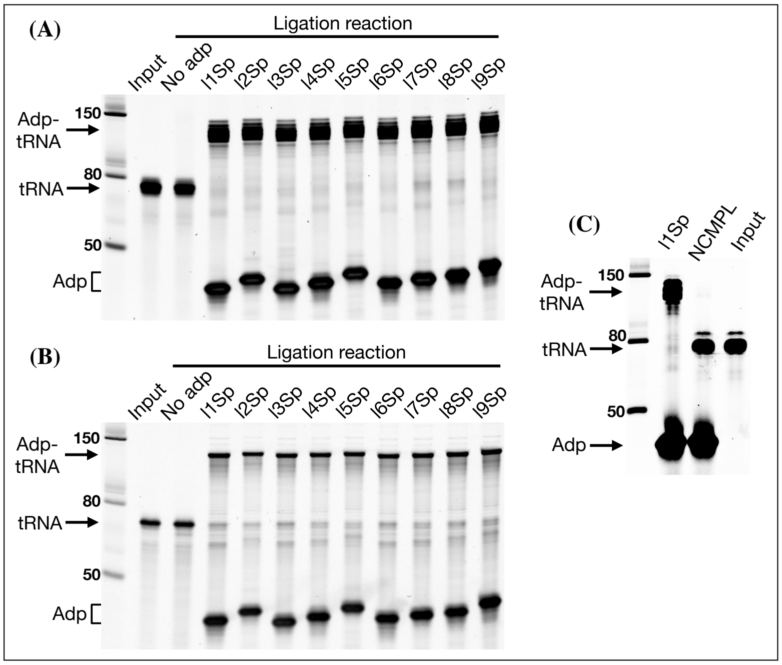

Figure 2—figure supplement 4. Ligation efficiency of all the barcoded adapters is high and depends on splint complementarity. **(A)** Ligation reactions using deacylated purified human tRNA as substrate. **(B)** Ligation reactions using E.coli tRNA-Lys-CC oligo as substrate. **(C)** Comparing ligation using a tRNA-end complementary splint (l1Sp lane) vs. a non-complementary splint (NCMPL lane). For both ligations the l1Sp adapter was used. For the non-complementary splint ligation the two standard TGGN and GGN overhang generating splints were swapped by two splints generating CAAC and AAC overhangs.

bioRxiv preprint doi: https://doi.org/10.1101/2023.07.31.551363; this version posted March 6, 2024. The copyright holder for this preprint (which was not certified by peer review) is the author/funder, who has granted bioRxiv a license to display the preprint in perpetuity. It is made available under aCC-BY 4.0 International license.

836

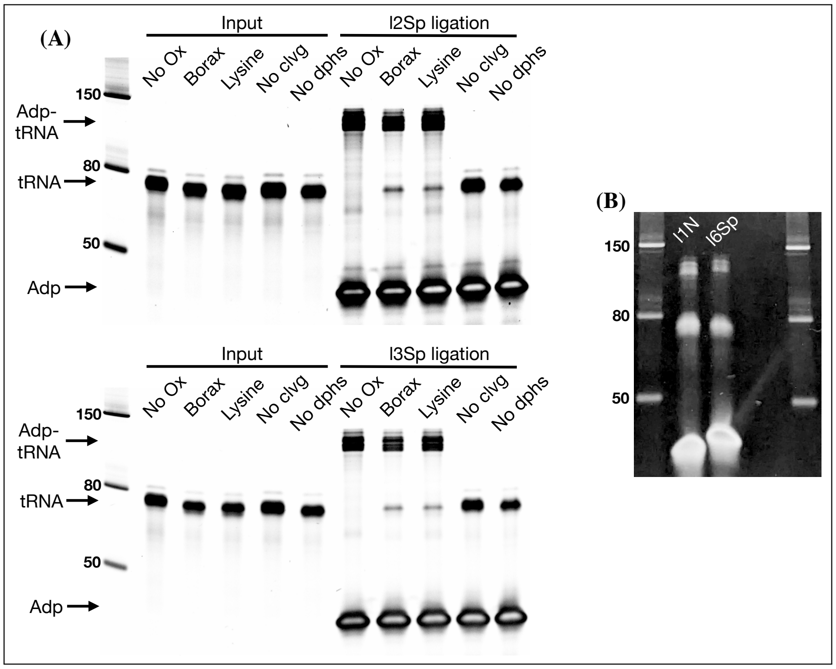

Figure 2—figure supplement 5. (A) Ligation test comparing the effect of RNA processing. Similar to **Figure 2**, panel E but with two different adapters. **(B)** The unligated tRNA that appears after tRNA is oxidized with periodate in panel A is refractory to further ligation. The unligated tRNA was gel purified from enough ligation reactions as shown in panel A to setup two new ligation reactions using either l1N pre-adenylated adapter for blunt end ligation or l6Sp for splint assisted ligation. For l1N, ligation was setup with 35 ng tRNA, 20 pmol adapter, 17.5% PEG-8000, 20% DMSO, 1xT4 RNA ligase buffer, 1 μL T4 RNA ligase 2 (truncated KQ) and 1 μL SuperaseIn. For l6Sp, the ligation was setup as described in the charge tRNA-Seq protocol.

bioRxiv preprint doi: https://doi.org/10.1101/2023.07.31.551363; this version posted March 6, 2024. The copyright holder for this preprint (which was not certified by peer review) is the author/funder, who has granted bioRxiv a license to display the preprint in perpetuity. It is made available under aCC-BY 4.0 International license.

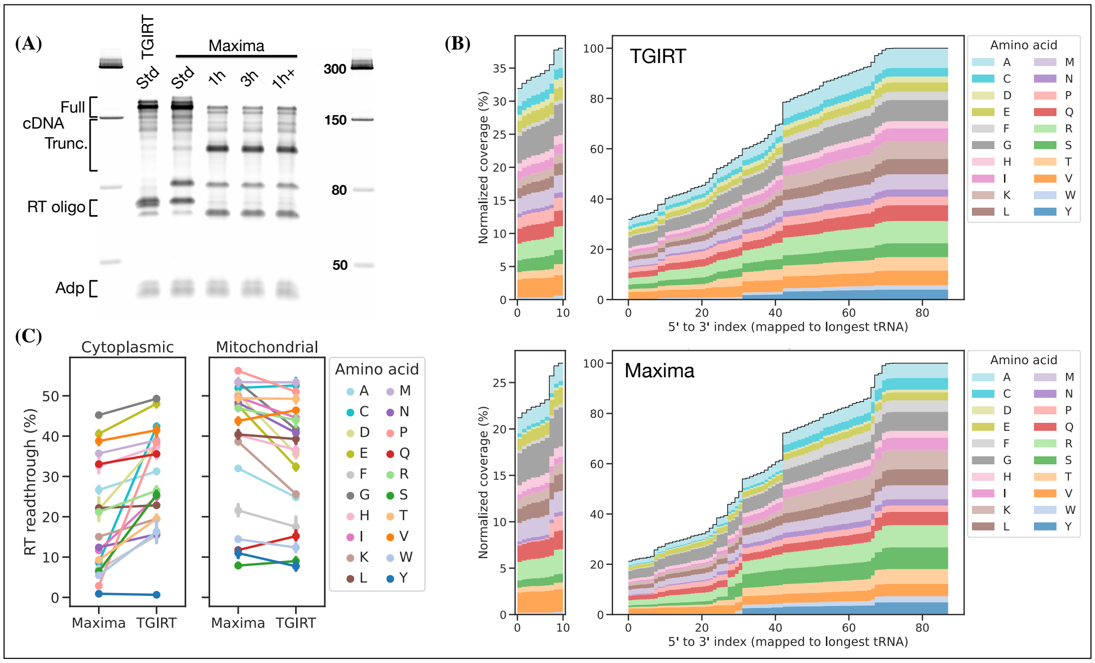

Figure 2—figure supplement 6. (A) The Maxima RT polymerase produces similar levels of full size cDNA as TGIRT-III under the standard (Std) tRNA-Seq RT-PCR conditions (42°C, 16 h, suggested by ***Behrens et al.*** (***2021***)). For Maxima, other incubation conditions tested are: 1 h at 60°C (similar to ***Lucas et al.*** (***2023***)), 3 h at 60°C and 1 h at 60°C followed by 15 h at 42°C (1h+). After RT-PCR, the RNA template was removed by NaOH hydrolysis, liberating the DNA adapter annotated on the gel. **(B)** Coverage plots for cytoplasmic tRNA transcripts grouped by cognate amino acid, comparing samples prepared with TGIRT-III or Maxima using standard incubation (42°C, 16 h). **(C)** Percentage of full length transcripts grouped by cognate amino acid (i.e. left side of plots in panel B). Errorbars are bootstrapped 95% confidence interval of the mean over the 7 individual samples, barcoded, pooled and used for RT-PCR template with both TGIRT-III and Maxima.

bioRxiv preprint doi: https://doi.org/10.1101/2023.07.31.551363; this version posted March 6, 2024. The copyright holder for this preprint (which was not certified by peer review) is the author/funder, who has granted bioRxiv a license to display the preprint in perpetuity. It is made available under aCC-BY 4.0 International license.

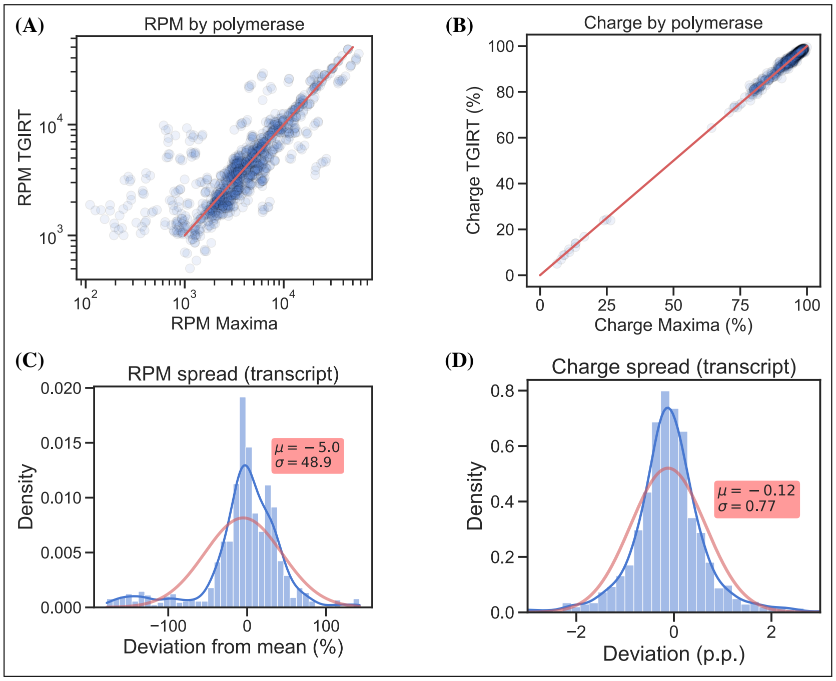

Figure 2—figure supplement 7. tRNA expression level measurements are sensitive to the choice of RT polymerase while charge measurements are not. **(A)** Comparing relative tRNA expression of TGIRT vs. Maxima. **(B)** Comparing tRNA charge of TGIRT vs. Maxima. **(C)** Density plot showing the percentage deviation of RPM measurements using TGIRT vs. Maxima, compared to the average of both. **(D)** Density plot showing the percentage point deviation of charge measurements using TGIRT vs. Maxima, compared to the average of both. Data is shown at transcript level for 7 individual RNA samples, barcoded, pooled and used for RT-PCR template with both TGIRT-III and Maxima. The red line in (A) and (B) is equality. Density plots are provided with kernel density estimate (KDE) in blue, normal distribution estimate in red and inserts with mean (μ) and standard deviation (σ). For density plots, a negative deviation is the result of RPM or charge readings being higher for TGIRT than for Maxima, and vice versa for positive deviations.

bioRxiv preprint doi: https://doi.org/10.1101/2023.07.31.551363; this version posted March 6, 2024. The copyright holder for this preprint (which was not certified by peer review) is the author/funder, who has granted bioRxiv a license to display the preprint in perpetuity. It is made available under aCC-BY 4.0 International license.

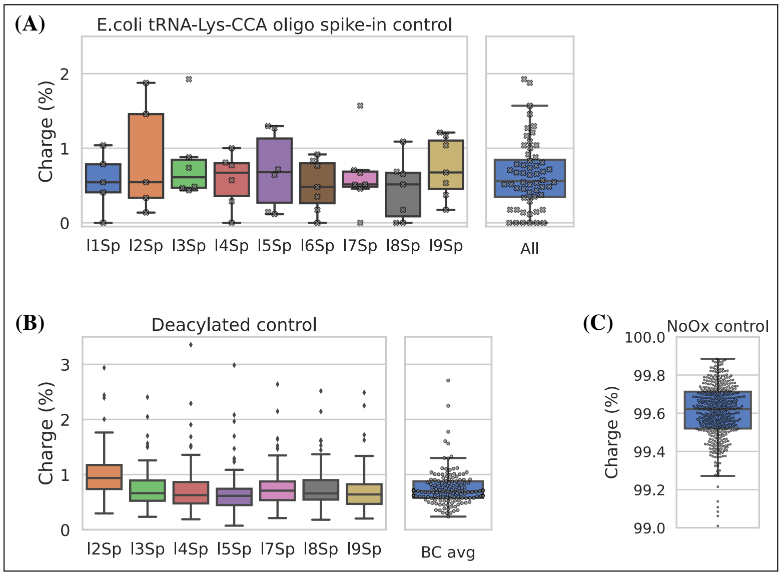

Figure 2—figure supplement 8. Charge tRNA-Seq control samples and spike-ins validate the method. **(A)** Cleavage of the 3' adenosine on spike-in oligo is near complete and similarly measured across adapters. Using the E.coli tRNA-Lys-CCA oligo as a spike-in control to monitor completion of the Whitfeld reaction. If complete, 100% E.coli tRNA-Lys-CC should be produced and thus appearing as 0% charged. Each dot represents one sample spiked with E.coli tRNA-Lys-CCA oligo before the Whitfeld reaction and processed using the charge tRNA-Seq processing described in the method section. **(B)** Aminoacylation level of human tRNA transcripts after undergoing deacylation by incubation at 45°C for 4 h in 1 M lysine (pH=8). Mitochondrial tRNA^{fMet} was excluded because formylated amino acids are known to be highly resistant towards deacylation (***Schofield and Zamecnik, 1968***). **(C)** Aminoacylation level of tRNA transcripts from four samples receiving sham oxidation (NaCl) during the Whitfeld reaction.

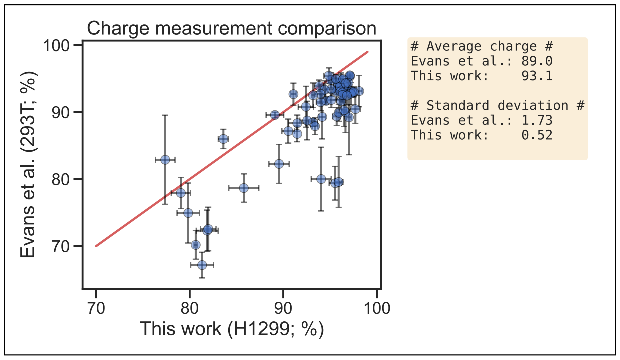

Figure 2—figure supplement 9. Charge measurement comparison between H1299 at baseline (this work) and 293T at baseline (***Evans et al., 2017***). Each point is an isodecoder shared by both datasets, errorbars are plus/minus one standard deviation of the sample replicates and the red line is equality. H1299 charge measurements with four replicates also displayed in **Figure 2**, panel F. 293T charge measurement with six replicates, from NCBI Geo database (accession GSE97259, supplementary file). In right text box, the average charge and the average replicate standard deviation, across all isodecoders.

bioRxiv preprint doi: https://doi.org/10.1101/2023.07.31.551363; this version posted March 6, 2024. The copyright holder for this preprint (which was not certified by peer review) is the author/funder, who has granted bioRxiv a license to display the preprint in perpetuity. It is made available under aCC-BY 4.0 International license.

841

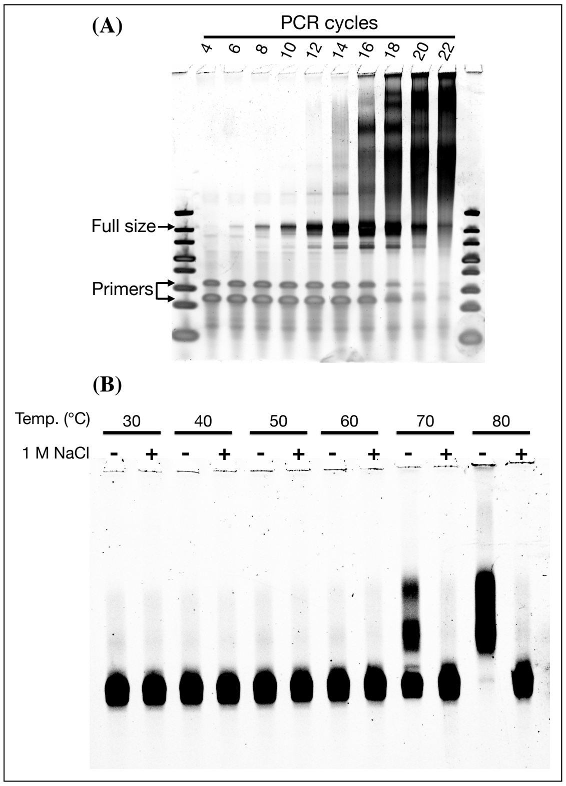

Figure 2—figure supplement 10. (A) The specificity of the final library PCR step (attaching Illumina P7 and P5 sequences) deteriorates with increasing product-to-primer ratios, probably due to high tRNA homology and PCR crossover (***Holcomb et al., 2014***). **(B)** tRNA-Seq DNA library reannealing is inhibited by high salt concentrations. A gel purified charge tRNA-Seq DNA library was resuspended in TBE buffer and incubated 30 min at different temperatures with or without 1 M NaCl.

bioRxiv preprint doi: https://doi.org/10.1101/2023.07.31.551363; this version posted March 6, 2024. The copyright holder for this preprint (which was not certified by peer review) is the author/funder, who has granted bioRxiv a license to display the preprint in perpetuity. It is made available under aCC-BY 4.0 International license.

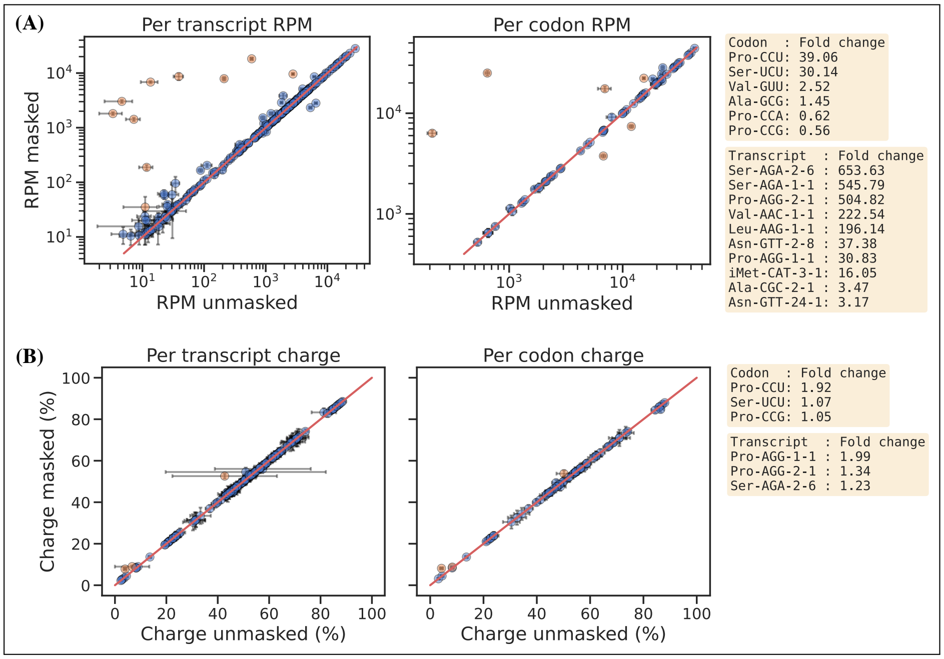

Figure 3—figure supplement 1. (A) Reference masking effect on RPM levels per transcript (left) and per codon (right). Transcripts showing > 3, and codons showing > 1.4, fold increase or decrease upon reference masking are colored orange and annotated on the right side of the plot. **(B)** Reference masking effect on charge levels per transcript (left) and per codon (right). Transcripts/-codons showing > 1.05 fold increase or decrease upon reference masking are colored orange and annotated on the right side of the plot.

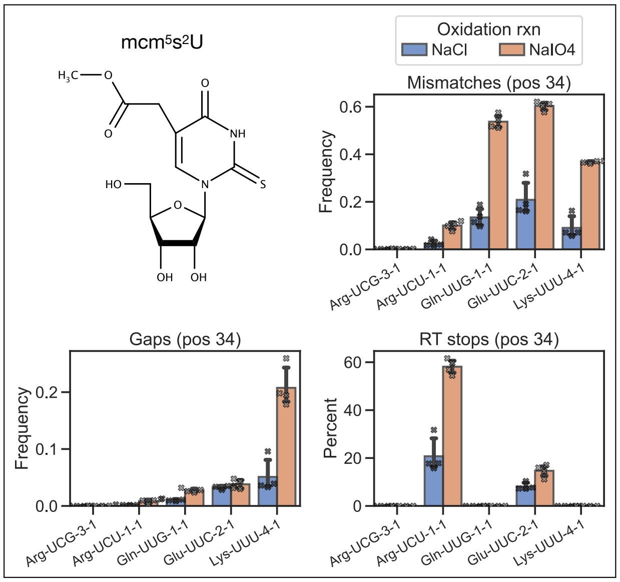

Figure 3—figure supplement 2. Mismatch frequency, gap frequency and RT stop percentage is increased upon periodate oxidation for transcripts known to be 5-methoxycarbonylmethyl-2-thiouridine (mcm^{5}s^{2}U) modified. The mcm^{5}s^{2}U modification has been shown to be present on the first anticodon nucleoside (position 34) in human tRNA Lys-UUU, Gln-UUG, Glu-UUC and Arg-UCU, while absent in the similar tRNA Arg-UCG (*Lentini et al., 2018*).

bioRxiv preprint doi: https://doi.org/10.1101/2023.07.31.551363; this version posted March 6, 2024. The copyright holder for this preprint (which was not certified by peer review) is the author/funder, who has granted bioRxiv a license to display the preprint in perpetuity. It is made available under aCC-BY 4.0 International license.

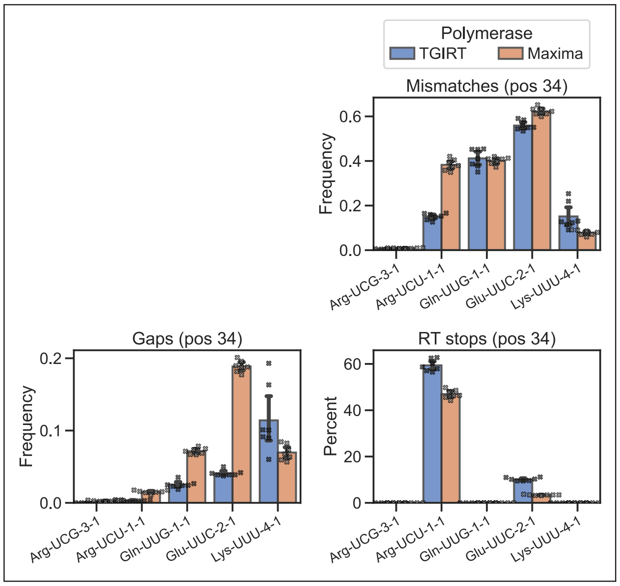

Figure 3—figure supplement 3. Polymerase dependent mismatch frequency, gap frequency and RT stop percentage for the mcm^{5}s^{2}U modified position. Comparing TGIRT vs. Maxima RT polymerases, otherwise similar to ***Figure 3—figure Supplement 2***. Data is shown for 7 individual RNA samples that were submitted to the Whitfeld reaction, barcoded, pooled and used for RT-PCR template with both TGIRT-III and Maxima.

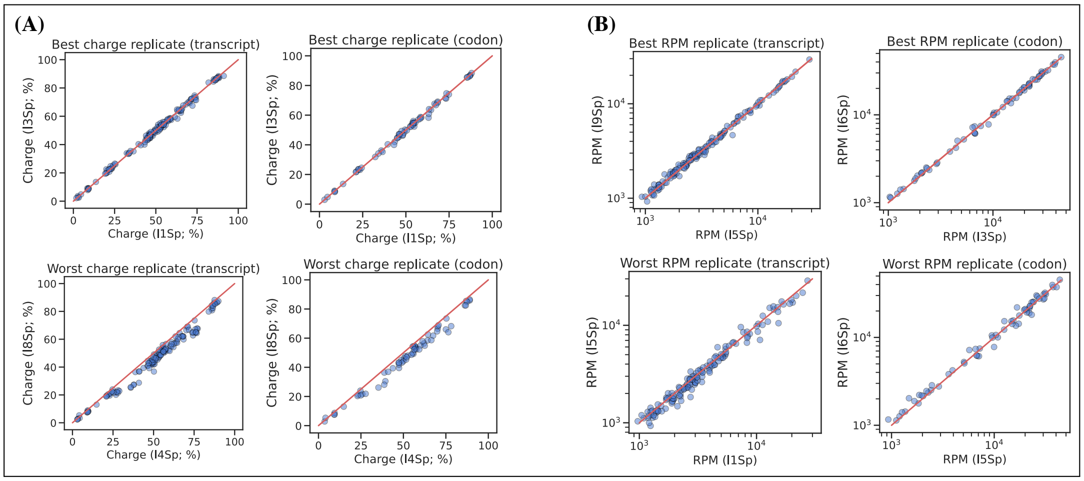

Figure 4—figure supplement 1. Best and worst pairwise comparisons between barcode replicates. Sorting pairwise comparisons between barcode replicates according to the sum of squared differences and showing the best and worst either at the transcript or codon level. **(A)** For charge levels, adapter l4Sp tends to overestimate charge. **(B)** For RPM levels. For all plots the red line is equality.

bioRxiv preprint doi: https://doi.org/10.1101/2023.07.31.551363; this version posted March 6, 2024. The copyright holder for this preprint (which was not certified by peer review) is the author/funder, who has granted bioRxiv a license to display the preprint in perpetuity. It is made available under aCC-BY 4.0 International license.

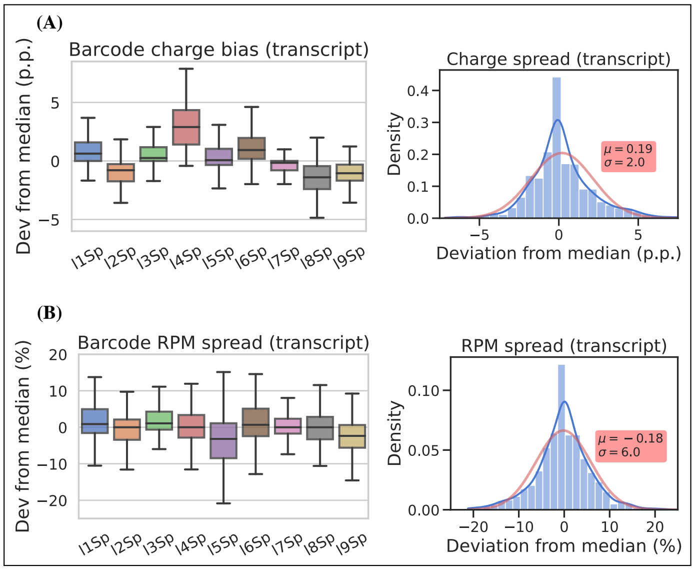

Figure 4—figure supplement 2. Similar to **Figure 4**, but at the transcript level.

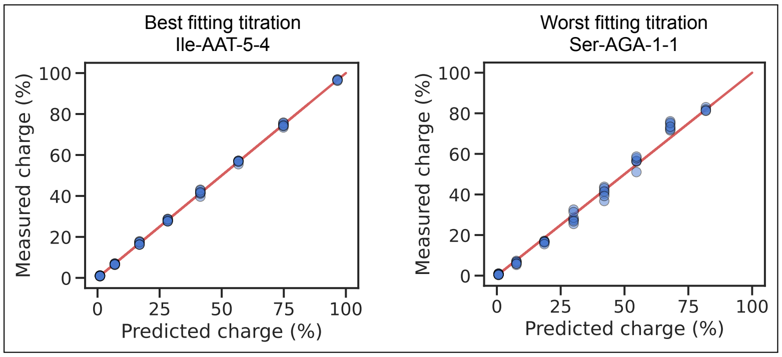

Figure 5—figure supplement 1. The best and worst transcript when ranked based on the sum of squared differences between the measured and predicted charge. Related to the representative (i.e. ranked as the median) transcript shown in **Figure 5**, panel B.

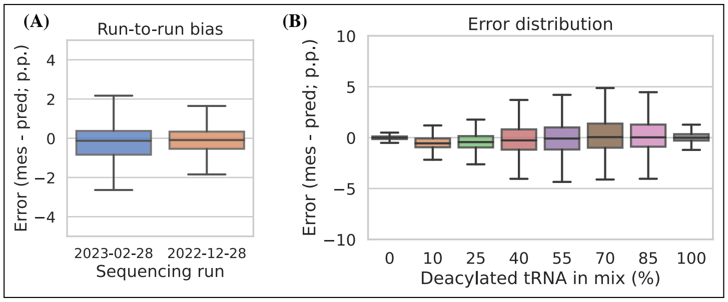

Figure 5—figure supplement 2. Charge titration prediction error binned by sequencing run and titration sample. **(A)** Run-to-run bias of two sequencing libraries independently prepared and sequenced on different days. **(B)** Error distribution binned by titration sample. In both panels, error is the percentage point difference between the measured vs. predicted charge for all transcripts in the bin.

bioRxiv preprint doi: https://doi.org/10.1101/2023.07.31.551363; this version posted March 6, 2024. The copyright holder for this preprint (which was not certified by peer review) is the author/funder, who has granted bioRxiv a license to display the preprint in perpetuity. It is made available under aCC-BY 4.0 International license.

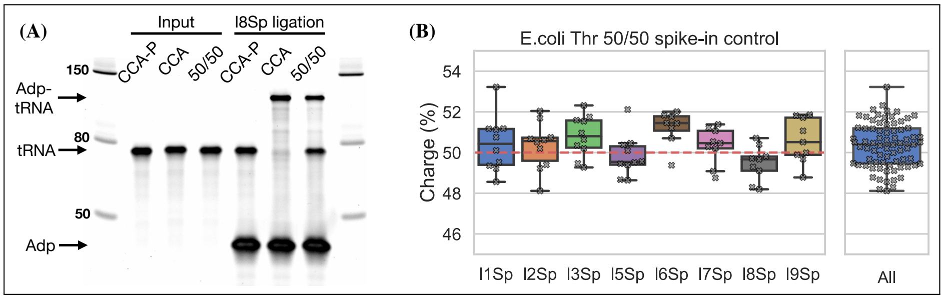

Figure 5—figure supplement 3. Spike-in control for 50% charge using the E.coli tRNA-Thr-CGT oligo. **(A)** Ligation between E.coli tRNA-Thr-CCA-Phos and l8Sp is completely blocked indicating ~100% 3' phosphorylation. CCA-P, E.coli tRNA-Thr-CCA-Phos. CCA, E.coli tRNA-Thr-CCA. 50/50, equal mix of CCA-p and CCA. **(B)** E.coli tRNA-Thr spike-in charge measured for samples prepared with an equimolar mix of E.coli tRNA-Thr-CCA-Phos and E.coli tRNA-Thr-CCA. Each dot represents a single charge tRNA-Seq sample. The red dashed line indicates 50% charge.

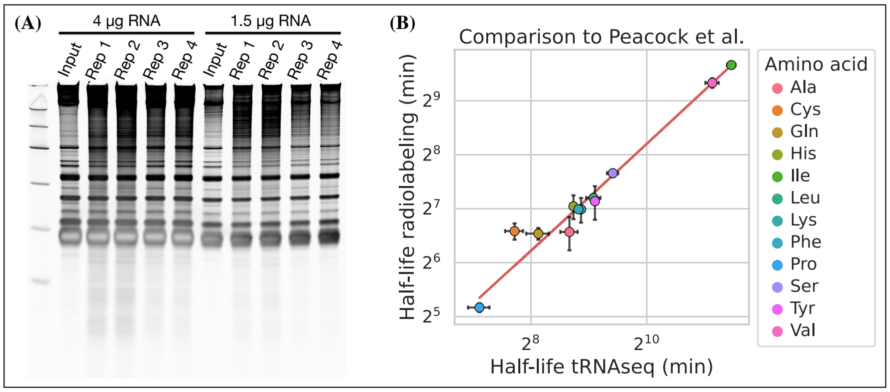

Figure 6—figure supplement 1. **(A)** RNA integrity after the last sample was taken (40 h) for the four replicates in the aminoacylation half-life experiment. **(B)** Comparison between aminoacylation half-life estimates grouped by amino acid from this study (tRNAseq) and measurements by ***Peacock et al.*** (***2014***) (radiolabeling). Errorbars are +/- standard deviations. A linear regression line is shown as a red line.

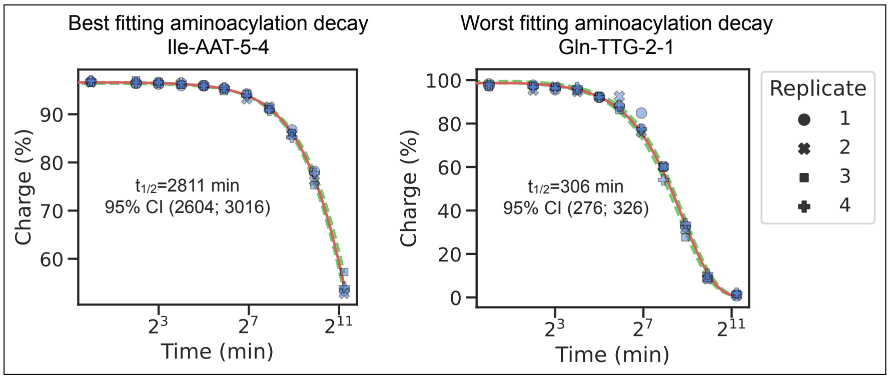

Figure 6—figure supplement 2. The best and worst transcript half-life estimates, ranked based on the sum of squared differences between the fitted decay function and the mean charge of the replicates. Related to the representative (i.e. ranked as the median) transcript shown in **Figure 6**, panel A.
